# CNS cancer immunity cycle and strategies to target this for glioblastoma

**DOI:** 10.18632/oncotarget.24896

**Published:** 2018-04-27

**Authors:** Malaka Ameratunga, Niamh Coleman, Liam Welsh, Frank Saran, Juanita Lopez

**Affiliations:** ^1^ Drug Development Unit, Royal Marsden Hospital and The Institute of Cancer Research, Sutton SM2 5PT, UK; ^2^ Department of Neuro-Oncology, Royal Marsden Hospital and The Institute of Cancer Research, Sutton SM2 5PT, UK

**Keywords:** glioblastoma, immunotherapy, cancer-immunity cycle, checkpoint inhibitors, clinical trials

## Abstract

Immunotherapeutics have revolutionized the management of solid malignancies over the last few years. Nevertheless, despite relative successes of checkpoint inhibitors in numerous solid tumour types, success in tumours of the central nervous system (CNS) has been lacking. There are several possible reasons for the relative lack of success of immunotherapeutics in this setting, including the immune microenvironment of glioblastoma, lymphocyte tracking through the blood-brain barrier (BBB) into the central nervous system and impairment of drug delivery into the CNS through the BBB. This review utilizes the cancer-immunity cycle as a conceptual framework through which the specific challenges associated with the development of immunotherapeutics for CNS malignancies can be viewed.

## INTRODUCTION

The recent development of immune checkpoint inhibitors and the corresponding efficacy shown by inhibitors of the CTLA-4/B7 [1] and PD-1/PD-L1 checkpoints [2–7] in multiple tumour types has resulted in substantial investment by the pharmaceutical industry in clinical development of immunotherapeutics across tumour types and indications. Although the efficacy of inhibitors of the PD-1/PD-L1 checkpoint has been consistent across tumour types, the single agent activity of these drugs has been lacking in tumours of the central nervous system (CNS). In particular, several studies have shown less promising results in glioblastoma compared with other tumour types [8, 9]. Glioblastoma, however, poses unique challenges to the immunotherapy treatment paradigm, as traditionally the CNS has been regarded as an immune-privileged site [10]; the frequent concomitant administration of immunosuppressive medications such as corticosteroids in this patient population is an additional consideration. Although these recent trials have cast doubts over the role, if any, of immunotherapeutics in CNS malignancies, they may also serve as an opportune time to evaluate the nuances of the emerging biology surrounding the cancer-immunity cycle and the specific challenges relating to drug development in primary brain tumours.

The cancer-immunity cycle was first proposed by Chen and Mellman [11] as a paradigm for the interaction between the immune system and cancer. They argue that a series of step-wise events must occur for effective anti-tumour immunity and coined the cycle to describe these events. Cancer cells and cancer cell death initially results in the release of neoantigens, which are then presented to dendritic cells. Priming and activation subsequently occurs, leading to trafficking of T cells to tumours, and subsequent infiltration of effector cells into tumours. There is then recognition of cancer cells by effector T cells, which results in cancer cell death which reiterates the cycle. This review evaluates the challenges of developing successful immunotherapeutics for glioblastoma through the lens of the cancer-immunity cycle. We initially describe the current understanding of the immune system in the central nervous system and subsequently address unique aspects of the immune system in the brain. We then describe current clinical development of CNS immunotherapeutics and the relative lack of efficacy of immune checkpoint inhibition to date. Finally, we provide a conceptual framework through which the development of effective immunotherapeutic strategies in the CNS can be viewed, and specific considerations for clinical trial design for CNS immunotherapeutics.

### The immune system and the brain – biological challenges and immune privilege

Historically, the CNS has been considered an immune-privileged site for a triumvirate of reasons [12]. Firstly, histological absence of observable lymphatics disputed lymphatic circulation in the brain, theoretically impeding functional immunity. Secondly, the blood-brain barrier (BBB) has been a major limitation since it was first described by Paul Ehrlich in the late 19^th^ century [13]. The BBB comprises a physical barrier due to complex tight junctions between adjacent endothelial cells, which requires transcellular passage of molecules trafficking into brain tissue compared to typical paracellular trafficking in other tissue sites [14]. Practically this results in limited penetration of antibodies, immune mediators and immune cells through the BBB from the systemic circulation into the CNS [15]. The third pillar of immune privilege was the disparity between the CNS immune system compared to the rest of the body, “apparent immune absence”, supported by observations such as the paucity of dendritic cells in the brain parenchyma [16], the seminal work of Lampson demonstrating the lack of major histocompatibility complex (MHC) class I on neuronal and glial tissue, the relative paucity of MHC class II expression in resections of brain tumour patients [17] and the tight regulation of the expression of T cell co-stimulatory molecules within the brain [18]. A large body of emerging work is now challenging the traditional assumptions underlying this concept of relative CNS immune privilege with good evidence indicating that the CNS is both immune competent and actively interacts with the peripheral immune system.

### Challenging lymphatic circulation as a pillar of immune privilege

**Figure 1:**
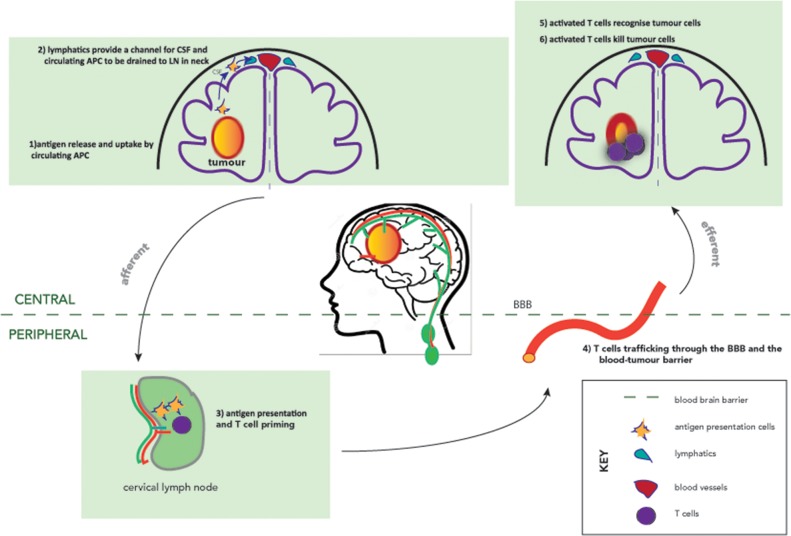
The afferent and efferent arms of the CNS immune system Dashed line indicates the blood-brain-barrier. Lymphatics are shown in green, and vasculature in red. Antigen release triggers recognition of antigens by antigen presenting cells, which are channelled via CNS lymphatics to the cervical lymph nodes. Antigen presentation and T cell priming occur peripherally in the cervical lymph nodes before trafficking back to the CNS to recognise and kill tumour cells.

Firstly, we now have clear evidence of lymphatic circulation within the brain [19]. Louveau and colleagues used sensitive imaging techniques to neatly show that the cerebrospinal fluid circulation leads to lymphatic drainage of the brain via the cervical and nasal lymphatics [20] suggesting that immune cells and tumour antigens may pass through the cerebrospinal fluid to the draining cervical lymphatics to meet with the antigen processing and presenting machinery and thereby stimulating the development of a systemic immune anti-tumour response (Figure 1). Although naive antigen-inexperienced T cells tend not to enter the healthy CNS and remained located in perivascular, subarachnoid, or meningeal spaces [21], activated CNS-specific CD4^+^ T cells are able to apparently chaperone naive non-CNS-specific T cells across the BBB into the CNS [22].

### Challenging the BBB as a pillar of immune privilege

Importantly, it is increasingly recognised that the BBB is dynamic, with its phenotype developing from complex cell-cell interactions from adjacent astrocytes [14, 23] and with its permeability varying based upon the functional requirements of signalling systems in the brain. For instance, the fenestrated endothelial wall at the hypothalamus allows diffusion of hormones into the systemic circulation, whereas the absence of the BBB at the area postrema allows relative free perfusion of molecules from the blood into brain tissue [13]. Of particular relevance in brain tumours, the tight junctions of the BBB can be disrupted in the setting of cerebral oedema [24], pro-inflammatory cytokines, such as interferon-γ and tumor necrosis factor-α [13], anatomical disruption from direct tumour extension, as well as downregulation of tight junction proteins such as claudins 1,3 [14, 25]. These observations gel with histopathological findings in brain tumour series, which have consistently demonstrated significant quantities of infiltrating immune cells in glioblastoma specimens, both macrophages but also CD4^+^ and CD8^+^ lymphocytes [26], as well as dynamic markers of the immune response such as PD-L1 [27].

Taken together, these factors demonstrate that the BBB is a relative rather than an absolute barrier, when considering implications for the trafficking of immune cells or the delivery of cancer therapeutics.

### Challenging apparent immune absence as a pillar of immune privilege

Finally, although there are definite differences in the immune system of the brain compared to other sites, this does not definitively preclude functional CNS immunity. Systemically, it is widely recognised that the critical components of the antitumour immune response are cytotoxic T cells and the adaptive immune system, and that overactivation of the innate immune system can paradoxically promote tumorigenesis [28]. Nevertheless, some degree of innate immune activation is a requisite for functional anticancer immunity. Critical components of the systemic anticancer immune response include immune recognition cells such as dendritic cells, immune effector cells such as cytotoxic CD8^+^ T cells and the supporting apparatus of CD4^+^ helper T cells. In the brain, microglia serve as the functional antigen presenting cells, having been shown by sensitive assays to avidly express MHC class II molecules, particular in the setting of inflammation, and are now thought to be able to directly present tumour antigens to T cells within the brain [29–31]. Although preclinical models of healthy mice suggested that the CNS parenchyma lacks a potent innate immune response [32], resident microglia are able to recognize “pathogen associated molecular patterns” and “danger associated molecular patterns”, which include heat shock proteins, uric acid, high-mobility group box 1 protein (HMGB1), and other structures available during tissue damage, inflammation and cell death [33]. Heat shock proteins released from tumor cells may be particularly effective chaperones for tumor-specific peptide antigens and may both activate dendritic cells and serve as antigen couriers [34, 35]. Thus, there appears to be sufficient innate immune system activation in the CNS to generate an antitumour immune response.

It has been challenging to identify how the innate immune system activates the adaptive antitumour immune response in the brain, but preclinical models suggests that activated dendritic cells carry antigens and transit to the cervical lymph nodes where a systemic immune response is stimulated [36]. Additionally CNS-derived soluble tumour antigens may directly drain to the lymphatics where they are presented by peripheral antigen-presentation machinery [37].

A CNS-specific T-cell trafficking programme is yet to be identified, but preclinical work in auto-immune murine models suggests that activation of T cells within the cervical lymph nodes have a direct role for the neuro-inflammation seen [38]. Three potential immune entry sites into the CNS have been described, localizing to the superficial leptomeningeal vessels, parenchymal vessels and the choroid plexus [39]. In agreement with these findings, immune cell infiltrates are found in tumor tissue derived from brain cancers consisting of both macrophages and CD4^+^ and CD8^+^ lymphocytes [26, 40]. Furthermore, antibodies are able to penetrate into the CNS, albeit at lower concentrations than in the systemic circulation [41], providing evidence of the humoral component of the adaptive immune system in the brain. These factors suggest that there is a functional cellular and humoral immune response in the brain, the key components of which are demonstrated in Figure 1. Counteracting this functional adaptive immunity is the increasing recognition of a particularly immunosuppressive tumour microenvironment in the archetypal primary CNS tumour, glioblastoma.

The immunosuppressive tumour microenvironment of glioblastoma has been well documented [42] and characterized by the myriad anti-inflammatory cytokines secreted by glioma cells. Cytokines such as tumor growth factor-β (TGF-β), interleukin (IL)-6, IL-10 and prostaglandin E2 actively suppress the expression of MHC on microglia, thereby limiting antigen presentation and diminishing the cytotoxic T cell response [43, 44]. The infiltrating T cell population is over-represented by regulatory T cells (Tregs) [45], which are regulated by factors such as the enzyme indoleamine 2,3-dioxygenase (IDO) [46] and serve functionally in brain tumors to suppress the immune system [47]. This diminished response is further exacerbated by the promotion of the alternative M2 macrophage phenotype in glioblastoma [48]. There is a substantial body of literature demonstrating that phenotype switching of tumour-associated macrophages from M1 to M2 promotes tumorigenesis in diverse ways [48]. In glioblastoma, the presence of M2 macrophages has been correlated with increasing histological grade, which is thought to be driven in some part by tumoral expression of macrophage colony-stimulating factor [45, 49]. Thus, the development of clinically efficacious immunotherapeutics in the brain must both consider the unique aspects of the CNS immune system and the historical pillars of immune privilege as well as offsetting contribution of the immunosuppressive microenvironment in glioblastoma.

### Current clinical developments in CNS immunotherapeutics

The use of the PD-1/PD-L1 immune checkpoint inhibitors to unleash the T cell response has been most studied immunotherapeutic strategy in glioblastoma, but has proved mostly disappointing in single agent studies presented thus far [8, 50, 51] (Table 1). Checkmate-143 was a Phase 3 study exploring nivolumab in comparison to bevacizumab in the setting of recurrent glioblastoma and demonstrated a tolerability profiles consistent with observations in other tumor types. Disappointingly however, CheckMate-143 did not meet its primary endpoint of improved overall survival, as presented by Reardon et al at World Federation of Neurooncology Societies 2017 with lower documented response rates in the nivolumab arm in spite of a hint of more durable responses in the responding patients [8, 52].

**Table 1:** Reported results of single agent checkpoint inhibitors trials in recurrent glioblastoma

Registration number	Treatment	Overall response rate^*^ (%), (N)	Comments
NCT02017717	Nivolumab	8% (n=153) [8]	Longer duration of response (11.1 mo compared to 5.3 mo for bevacizumab). Median PFS 1.5 months. 12-month OS 42%.
NCT02054806	Pembrolizumab	4% (n=26) [53]	Median OS 14.4 months. Median PFS 2.8 months.
NCT02336165	Durvalumab	13.3% (n=31) [9]	12-month OS 44.4% 6-month OS 59.0% 6-month PFS 20.0%

^*^ Overall response rate according to RANO criteria.

PFS- progression-free survival.

OS- overall survival.

**Figure 2:**
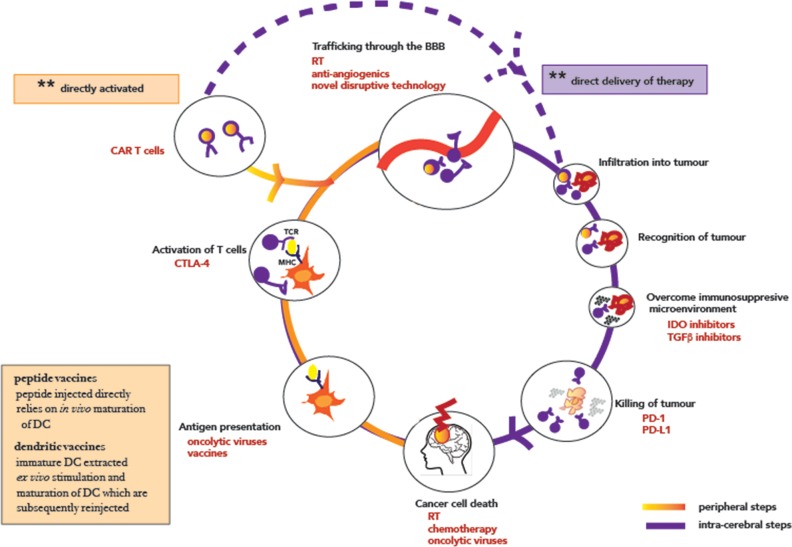
The cancer-immunity cycle in CNS malignancies T lymphocytes are shown in purple, with CAR-T modified T lymphocytes highlighted with a glow. The orange half of the circle marks out steps that can be targeted systemically, while the purple indicates steps that require intra-cranial delivery/mode of action. Abbreviations: CAR Chimeric antigen receptors; RT radiotherapy; CTLA-4 cytotoxic T-lymphocyte-associated protein 4; PD-1 Programmed cell death protein 1; PD-L1 Programmed death-ligand 1; IDO Indoleamine-pyrrole 2,3-dioxygenase; TGF-β Transforming growth factor beta.

Of note however, are the case reports of therapeutic successes in specific pediatric patients with biallelic mismatch repair deficiencies [54] suggesting that these antibodies do cross the BBB and penetrate into the tumour microenvironment, and are able to release a tumour specific cytotoxic T cell response. Given that these patients have hypermutated tumours with significantly high mutational load and therefore a significant immunogenic burden and thus a larger repertoire of tumor antigen-specific T cells [55, 56], the inclusion of a selected subset of glioma patients with high mutational burden into clinical trials of checkpoint inhibitors is one strategy currently being pursued (for example, in NCT02628067). As a population however, the mutational load in primary malignant brain tumours is low, approximately 10-fold lower than in melanoma and lung cancer [57, 58] with the mutational load being associated with tumour grade [59]. And although the currently available standard treatments of radiation and temozolomide are themselves mutagenic [60], and one may extrapolate that in cells that survive, the neoantigen load is likely to rise, thereby diversifying epitopes available for recognition by T cells, this has been insufficient in isolation to stimulate an adaptive immune response as demonstrated by the limited sensitivity to single agent immune checkpoint inhibition in the recurrent setting (Table 1). As such, consideration of other nodes in the CNS immunity cycle to be targeted with combinatorial strategies are urgently needed and discussed in detail in the following sections (Figure 2).

### The CNS cancer-immunity cycle- a framework for immunotherapeutic strategies in CNS tumour

#### Cancer cell death- DNA damaging agents and immunogenic cell death

Initiating the cancer immunity cycle is cell death and immunogenic cell death refers to activation of the immune system by apoptotic cells or pre-apoptotic cells resulting in tumor cell death [61]. DNA damaging agents including radiation and temozolomide can cause immunogenic cell death and the release of danger signals including “damage-associated molecular patterns” that stimulate the recruitment of APCs where they process and present tumour neoantigens, thereby priming an adaptive immune response [62]. It is worth noting that to date there does not appear to be any evidence that immunogenic cell death is affected by mutational load [62]. In preclinical murine glioma models, combined PD-1 blockade and stereotactic radiosurgery (SRS) have been shown to improve antitumor immunity and produce long-term survivors [63, 64] and this concept is now in early clinical testing in patients with malignant brain tumours. The focus on augmenting immunogenic cell death in glioblastoma to negate the limited single agent efficacy of PD-1 inhibition is translating into ongoing early phase clinical trials. Sahebjam et al recently presented preliminary findings from one such phase I study evaluating the concomitant use of hypofractionated SRS, pembrolizumab, and bevacizumab for recurrent, high-grade gliomas noting that all patients tolerated the regimen, and and an impressive durable response rate (response for ≥ 6 months) of 53% was noted [65, 66]. Numerous other combination trials of immunotherapy in combination with DNA damaging agents for CNS malignancies are ongoing including with temozolomide (e.g. NCT02311920), radiotherapy (NCT02617589, NCT02336165) and the combination of temozolomide and radiation (NCT02667587).

#### Antigen presentation- oncolytic virotherapy and vaccine strategies

Cell death can kickstart the cancer-immunity cycle in the brain by activating the adaptive immune system via antigen presentation. There are several complementary therapeutic strategies that are focussing upon this component of the cancer-immunity cycle in the brain. Oncolytic virotherapy makes use of non-pathogenic viruses to selectively invade or specifically express proteins in brain tumor cells that can directly kill cancer cells or otherwise stimulate an immune response, therefore marrying the concepts of immunogenic cell death with antigen presentation. The oncolytic polio virus utilizes the aberrant expression of the poliovirus receptor, CD155, in solid tumours to mediate viral cell entry [67]. In humans, infection of tumor macrophages and dendritic cells is sublethal and eventually leads to induction of MHC class II expression and the stimulation of a tumor antigen-specific T cell response [67, 68]. A Phase I clinical trial of a poliovirus chimera, PVSRIPO for recurrent glioblastoma showed that this approach was safe, with initial promising results, with 10 out of the initial 13 patients treated still alive at the end of the trial [69]. To overcome the attenuated immune responses within the brain, groups are attempting to engineer virotherapy with inducible inflammatory cytokines, for example the Ad-RTS-hIL-12, an inducible adenoviral vector that expresses IL12 in the presence of an orally-administered activator ligand, veledimex. This early phase trial showed evidence of systemic increases of IL-12, IFNγ as well as increased number of CD8+T-cells in circulation, with an impressive 100% 6-month survival for the 13 patients thus far [70]. The challenge here is that virotherapy for brain tumors relies heavily on viral migration to the tumor site and has mostly been explored by intratumoural injection which is not always achievable. Efforts are therefore underway to explore the feasibility of systemic intravenous delivery approaches to overcome this (e.g. REOGLIO ISRCTN70044565).

Apart from tumour cell lysis mediated by oncolytic viruses, there are complementary methods of targeting antigen presentation in the brain. The identification of a growing number of potentially unique immunoreactive tumor-associated antigens expressed by human gliomas make cancer vaccines including peptide, dendritic cell, tumor cell, and neoantigen vaccines a very exciting strategy. Moreover, this approach can be utilized peripherally, bypassing the logistical challenges of delivering therapeutics directly intracranially. Peptide vaccines induce a T-cell response at the tumor site by releasing peptides specific to tumor-associated antigens. These are commonly coupled with carrier proteins and adjuvants, are taken up by APCs and presented on the cell surface by MHC molecules. APCs navigate the lymphatic system to prime T-cells, which then recognize the tumor cell from its antigen [71]. Glioblastoma represents an attractive therapeutic target for peptide vaccination as the unique epidermal growth factor receptor (EGFR) variant, EGFRvIII,is expressed in approximately 30% of patients with glioblastoma [72]. The most advanced therapeutic candidate peptide vaccine is rindopepimut, which targets a neoepitope created by a 13 amino-acid sequence unique to EGFRvIII, chemically conjugated to KLH which serves as an immune adjuvant [73]. Although initially heralded as a major breakthrough on the back of positive early phase studies [74], recent published large phase three studies have failed to show a survival benefit and argue against rindopepimut's efficacy [75], and this may be largely due to the heterogenous nature of glioblastoma. To address this issue of heterogeneously-expressed tumor-associated antigens, multi-peptide vaccine strategies such as the IMA950 vaccine which contains 11 human leukocyte antigen (HLA)-restricted tumor-associated peptides are being explored with some initial hints of benefit, particularly in a sub-group of patients with marked injection site reactions [76]. Other candidate peptide vaccines are also showing initial promise in early phase clinical trials [77, 78] and the results of larger studies are eagerly waited.

The alternative vaccine strategy is of dendritic cell vaccination. Instead of injecting a peptide that is presented to an APC, autologous dendritic cells sourced from peripheral blood monocytes are primed with tumour lysate from the patients’ own tumour in the presence of growth factors such as interleukin-4 and granulocyte macrophage colony stimulating factor, [79]. Immature dendritic cells can uptake and process tumour-associated antigens, and mature *ex vivo*, thus becoming capable of proper antigen presentation for T-cell recognition in a MHC-restrictive manner [80]. These pools of dendritic cells are subsequently autologously transplanted into patients. Studies performed in glioblastoma patients have typically involved injection intradermally [79, 81] in proximity to the draining cervical lymph nodes, or occasionally in patients with Ommaya reservoirs, directly into the cerebrospinal fluid [81]. In these studies, although unarguable clinical benefit could not be observed, there was clear evidence of increases in tumour-lysate specific T cells in the periphery [81] and tumour lysate specific memory T cells and cytotoxic T cells intratumorally [79]. One example is the ICT-107 autologous dendritic cell vaccine pulsed with six tumor-associated antigens for which ten-year follow-up data is available for the initial Phase I vaccine trial. 19% of 16 patients remained disease free for 8 years with a median overall survival of 38.4 months [82]. These durable responses have fueled combination studies with checkpoint inhibitors which are ongoing (for example NCT02529072).

#### T cell activation

Antigen presentation is followed by T cell activation in the cancer immunity cycle, which represents another potential target of immunotherapeutic strategies in the brain. The inhibitory cell surface protein CTLA-4 primarily regulates the amplitude of the early stages of T cell activation [83] and is expressed solely by T-cells localized primarily within secondary lymphoid tissues. It binds preferentially to CD80/CD86 on the surface of APCs, thus preventing their binding to the T-cell co-stimulatory receptor CD28, leading to decreased T-cell activation and proliferation in the context of antigen-presenting MHC class [84–87]. CTLA-4 also contributes to immune modulation by enhancing the suppressor functions of Tregs [88].

The combination of anti-CTLA-4 plus anti-PD-1 has demonstrated encouraging activity in preclinical murine models of orthotopic transplanted gliomas [45, 63, 64, 89, 90], however this has failed to translate to substantial clinical benefit (8). In the Phase I CheckMate-143 study, 90% of patients who received combination therapy had grade 3 or 4 treatment-related adverse events, and 50% of patients in that arm had to discontinue treatment early due to intolerability leading to the exclusion of this combination in the subsequent phase II/III study [52]. In patients with an overall poor prognosis, this limited efficacy combined with significant toxicity is unacceptable and as such, needs tweaking to deliver tangible clinical benefits to patients. One approach to minimize the risk of increased systemic toxicity from these combination is to use intra-tumoral delivery of anti-CTLA-4 following the resection of the recurrent glioblastoma which is currently ongoing (NCT03233152).

#### Lymphocyte-trafficking into the CNS: BBB

Following T cell activation, the CNS cancer immunity cycle needs to consider trafficking into the CNS and crossing the BBB. The therapeutic strategy most advanced in glioblastoma that may theoretically affect the BBB is anti-angiogenic therapy. Although, initially uptake of anti-angiogenics was met by optimism due to unprecedented response rates [91], subsequent large randomised trials have failed to demonstrate evidence of benefit [92, 93] and a large meta-analysis has shown no overall survival benefit for these agents [94]. Nevertheless, emerging data support a strong rationale for combining therapies targeting vessel normalization with immunotherapies [95]. In particular, abnormal tumour vasculature promotes the production of cytokines which preferentially recruit immunosuppressive lymphoid populations [95] and polarize tumor associated macrophages to the immunosuppressive M2 phenotype [48]. As such, combinations of anti-angiogenics together with checkpoint inhibitors are actively being pursued in early phase clinical trials (NCT02336165, NCT02337491). It is however, worth noting that glioblastoma is a highly invasive tumour, and that anti-angiogenic agents may paradoxically promote invasiveness [96, 97] thus impeding the efficacy of this combination.

Other ingenious out-of-the-box solutions are being explored to overcome the impediment of the BBB in drug delivery. Armed with the knowledge that some of the activity of radiotherapy in brain tumours is due to disruption of tight junctions and therefore vessel permeability [98], the hypotheses that low dose radiotherapy could increase drug delivery to the CNS was recently tested [99]. Preliminary results in a cohort of resected brain metastases patients has demonstrated substantially (~20x) higher tissue afatinib concentrations compared to plasma, thereby validating this hypothesis. Other viable strategies to disrupt the BBB undergoing clinical evaluation include the combination of microbubble injections with pulsed ultrasound, which has been shown to functionally disrupt the BBB on serial contrast-enhanced MRI [100]. These trials provide proof-of-principle that augmentation of drug delivery into the CNS could be achieved and is likely to be used in combination strategies soon.

#### Infiltration and recognition of tumour- adoptive cell therapy

Once lymphocytes have been trafficked to the tumour, the effector components of the immune system must infiltrate into the tumour and recognise the tumour to propagate the CNS cancer immunity cycle. One strategy targeting this component of the cycle is adoptive cell therapy. Instead of relying on the afferent of the neuro-immune system, adoptive cell therapy aims to engineer and directly activate T cells which are then able to home back to the tumour (Figure 2). This technology, first developed by Gross et al [101] utilizes a chimeric construct consisting of a single-chain variable fragment of a high affinity antibody recognizing a tumour antigen fused to one/multiple co-stimulatory domains that directly activate T cells (CAR-T) in a non-MHC restricted manner [101] They have exhibited striking activity in hematological malignancies and the first CAR-T therapy recently being approved by the FDA for use in relapsed B cell precursor acute lymphoblastic leukemia [102]. Efforts in solid tumours are ongoing (see Table 2), but suffer from lack of well described cell surface targets which are solely expressed on tumour cells and absent from normal tissue [103]. In some ways, glioblastoma is relatively fortunate compared to other solid malignancies, with the well described truncating EGFRvIII variant [72] exhibiting characteristics of an opportune target – high frequency aberration in target disease and absence in normal tissue. Consequently, CAR-T cells targeting this variant are undergoing clinical development [104]. The first-in-human Phase I study of CAR-T EGFRvIII cells demonstrated the safety of this approach, without evidence of off-target toxicity or cytokine release syndrome with one patient having stable disease at 18 months [105].

**Table 2:** Ongoing trials of CART cells in glioblastoma

NCT number	Tumour type	Target	Mode of delivery
NCT02331693	Advanced Glioma	EGFR	Systemic infusion (IV)
NCT02209376	Glioblastoma Multiforme	EGFRvIII	Systemic infusion (IV)
NCT02844062	Glioblastoma Multiforme	EGFRvIII	Systemic infusion (IV)
NCT01454596	Glioblastoma Multiforme	EGFRvIII	Systemic infusion with aldesleukin (IL-2) (IV)
NCT02937844	Glioblastoma Multiforme	PD-L1	Systemic infusion (three-day split) (IV)
NCT02575261	Glioma	EphA2	Systemic infusion (IV)
NCT02664363	Glioblastoma Multiforme	EGFRvIII	Systemic infusion (IV) Companion imaging study.
NCT01082926	Brain tumours	IL13Rα2	Intratumoral
NCT02208362	High grade glioma	IL13Rα2	Intratumoral
NCT02442297	Glioblastoma Multiforme	Her2	Intratumoral
NCT01109095	Glioblastoma Multiforme	Her2 (CMV specific T cells)	Systemic infusion (IV)

Other antigens being targeted in current clinical trials include Eph-A2 and IL13Rα2 (see Table 2). Preliminary results from a Phase I trial of a first-generation CAR-T cells targeting the glioblastoma tumor antigen IL13Rα2 reported safe intracranial delivery of the CAR-T cells with one particular patient exhibiting a 79% regression of recurrent tumour mass [106]. Building on this, a 2^nd^ generation CAR-T incorporating a 4-1BB (CD137) costimulatory domain and a mutated IgG4-Fc linker to reduce off-target Fc-receptor interaction is in testing with a dramatic transient clinical response in a patient with recurrent multifocal glioblastoma [107]. Two important lessons can be drawn from this study – firstly, the challenge of T cell homing as this patient did not respond to the initial intercavitary delivery of CAR-T cells, but responded dramatically when this was switched to an intra-ventricular mode of delivery. And secondly, despite the incredible radiological response, the patient relapsed with tumours that had significantly decreased IL13Rα2 expression suggesting that antigenic heterogeneity may be a significant hurdle to the success of this approach. Technical advances in cellular engineering may help overcome some of these challenges, for example a recent preclinical study has shown that trivalent CAR-T cells targeting commonly expressed glioma antigens including HER-2, IL13Rα2 and Eph-A2 can overcome tumour heterogeneity and target nearly all tumour cells in patient-derived xenograft models compared to bispecific or single-epitope targeting CARs [108].

#### Overcoming the suppressive immune microenvironment

Finally, for ongoing cell death to perpetuate the CNS cancer immunity cycle, the immunosuppressive microenvironment must be overcome. The challenge of the immunosuppressive microenvironment has been particularly highlighted by the early phase CAR-T trials. O’Rourke and colleagues found evidence of trafficking of CAR-T-EGFRvIII cells to regions of active glioblastoma, with antigen decrease in five of these seven patients who proceeded to surgery, but in all cases in situ evaluation of the tumour microenvironment demonstrated increased and robust expression of inhibitory molecular and infiltration by regulatory T cells after CAR-T-EGFRvIII infusion, compared to pre-infusion specimens [105]. As such, novel strategies targeting the immune microenvironment are urgently required. Components of the immunosuppressive microenvironment include Tregs, monocytes as well as signaling molecules, all of which could theoretically be targeted to enhance anti-cancer immunity in glioblastoma.

Given the prominence of the M2 macrophage phenotype in glioblastoma [49], strategies aiming to switch macrophage polarization are being explored. Preclinical models implicate the macrophage colony stimulating factor 1 receptor (CSF-1R) in macrophage/monocyte polarization to the pro-tumorigenic M2 phenotype and antagonists to this are in clinical testing (NCT02526017). Other signaling molecules including the phosphoinositide 3-kinase (PI3K) signaling pathway also have a role in directly polarization of macrophages to the M2 phenotype [109] and despite limited single agent activity of multiple PI3K pathway inhibitors in glioblastoma [110], these may have value combinatorially.

TGFβ, secreted by tumour cells in an autocrine loop is a potent immunosuppressive cytokine and inhibits the efficacy of immune effector cells [111]. A bispecific antibody targeting PD-L1 and TGFβR2 has shown preclinical evidence of enhancing antibody-dependent cellular cytotoxicity mediated by both PD-L1 and TGFβR2 preclinically [111] and is now in clinical trials including a glioma cohort of patients [112] (NCT02517398). Other ongoing trials include combinations with the TGFβR1 inhibitor galunersertib (NCT02423343).

The immunoregulatory enzyme IDO has been heavily associated with immune tolerance [113] and has been specifically associated with controlling the functional status of Tregs in response to inflammatory stimuli [46]. Inhibitors of this enzyme are amongst the most advanced novel immunotherapeutics in clinical development with multiple clinical trials ongoing in numerous tumour types including in glioblastoma (NCT02052648). Although single agent activity of IDO inhibitors have not been promising in solid tumours [114], recent reports of significantly higher response rates in combination with PD-1 inhibitors have prompted excitement [115] and this strategy may have utility in combinations for glioblastoma.

### Considerations for CNS drug development

In this review, we have presented a framework for understanding the CNS-cancer immunity cycle to effectively develop immunotherapeutics for CNS tumours. Table 3 summarises the components of the cancer immunity cycle and current strategies targeting these components. Rational strategies backed by strong preclinical data for combinations must be developed to optimize efficacy. Specific challenges unique to brain tumours must be considered. One of the major hurdles in developing preclinical insights is the lack of biologically relevant models for hypothesis testing. Moreover, although in other solid tumours sequential tumour biopsies are increasingly used to compress clinical development timelines and improve pharmacodynamic studies [116], given the relative importance of brain tissue and associated difficulty with tissue sampling, this strategy is simply not feasible in CNS tumours. Nevertheless, there are ways to combat this specific issue. Having optional research biopsy components in patients who are undergoing re-resections for clinical reasons can bypass this problem. Pharmacokinetic information can also be established with cerebrospinal fluid samples, which has previously added useful information to pharmacokinetic profiles [117].

**Table 3:** Current strategies targeting the cancer-immunity cycle in glioblastoma

Cancer immunity cycle component	Possible therapeutic strategy	Examples of current trials
Cell death	Combination with DNA damaging agents Combination with stereotactic radiosurgery	NCT02311920, NCT02617589, NCT02336165 NCT02667587 NCT02313272
Antigen presentation	Oncolytic viruses Vaccines	ISRCTN70044565 NCT02529072
T cell activation	Intratumoural CTLA-4 combination	NCT03233152
Lymphocyte trafficking	Combination with antiangiogenic agents	NCT02336165, NCT02337491
Infiltration and recognition of tumour	CART cells/ adoptive cell therapy	NCT02209376
Overcoming the suppressive immune microenvironment	Macrophage polarization Bispecific antibodies Immunoregulatory inhibitors	NCT02526017 NCT02517398 NCT02052648

Other specific challenges unique to the CNS include the BBB, which is an impediment to effective CNS penetration of numerous drugs. There are many approaches that that may mitigate this problem. Firstly, several trials are currently being performed on small molecule inhibitors, with the compound being delivered immediately in the pre-operative period prior to re-resection, thus allowing for a more substantial study of pharmacodynamic endpoints. Secondly, given a substantial component of the cancer-immunity cycle occurs peripherally, there is no reason why therapeutics targeting the periphery cannot have central activity.

There are also some unique clinical considerations in glioblastoma patients that can impede effective drug delivery and drug development. Many patients with brain tumours have uncontrolled seizures requiring numerous anti-epileptic medications. These represent a challenge in early phase clinical trials, as typically the use of such drugs is prohibited due to the uncertain pharmacokinetic profiles that they result in, particularly in the development of drugs predicted to be metabolized by the hepatic cytochrome p450 system. However, it is important to note that second and third generation anti-epileptic medications are typically not enzyme inducing and therefore limit the risks of adverse drug-drug interactions and eligibility for participation in early phase clinical trials.

Additionally, there is specific concern regarding the use of immunotherapeutics. A major impediment to effective *in vivo* activity in patients with primary brain tumours is the oft-needed baseline use of corticosteroids to control intra-cerebral edema. It is well known that corticosteroids diminish immune activity and therefore their presence at baseline could impair the robustness of any anti-tumour immune response. In this respect, combination strategies with drugs such as bevacizumab which may have a steroid sparing effect [118] may augment anti-tumour immunity. Moreover, if a response was nevertheless to occur, there remains concern that tumour flare may present with mass effect like symptoms, which can be quite significant in a patient population already suffering from cerebral edema, or auto-immune neurotoxicity. Caution must continue, though it is reassuring that most reported studies of checkpoint inhibitors in glioblastoma to date have not shown an adverse event profile substantially dissimilar to other solid tumours which mitigates the latter point [8, 119].

Finally, although the various immune combination strategies described in this review hold promise due to their underlying biological rationale, implementation of any of these strategies needs to take into account the cost of these technologies with a keen focus on the ultimate value delivered to be patients [120].

## CONCLUSION

In conclusion, despite the disappointing results of single agent immunotherapeutics to date, there remain reasons to be not only be optimistic, but excited. Understanding the CNS cancer immunity cycle provides a suitable framework upon which the various approaches and challenges to CNS drug development can be expounded and will be the foundation for the development of rational combination strategies to improve patient outcomes in this disease.
